# Study on the influence of family capital on Chinese adolescents’ subjective well-being

**DOI:** 10.3389/fpsyg.2022.989229

**Published:** 2022-08-24

**Authors:** Xiling Wu, Zhiyan Liu, Youchang Lin

**Affiliations:** ^1^School of Public Administration, SiChuan University, Chengdu, China; ^2^Guangdong Zhongda Management Consulting Group Co. Ltd.,, Guangzhou, China; ^3^School of Marxism, University of Electronic Science and Technology of China, Chengdu, China; ^4^College of Marxism, Chengdu University of Traditional Chinese Medicine, Chengdu, China

**Keywords:** family capital, school climate, academic achievement, adolescents’ subjective well-being, psychology

## Abstract

Subjective well-being (SWB) is an important part of positive psychology research. Compared with other countries and regions, Chinese adolescents’ well-being level is relatively lower. Under the guidance of ecological systems theory, this study is based on the survey data of PISA 2018, with 10,805 middle school students from four provinces and cities in China (Beijing, Shanghai, Jiangsu, and Zhejiang) as the research samples, and examines the theoretical model of the influence of family capital on adolescents’ subjective well-being by means of structural equation modeling, in which the effects of family capital, school climate and academic achievement on adolescents’ subjective well-being are discussed. The empirical results show that family capital directly and indirectly negatively influences adolescents’ subjective well-being through academic achievements, and indirectly positively influences adolescents’ subjective well-being through school climate. School climate directly positively affects adolescents’ subjective well-being and indirectly negatively influences adolescents’ subjective well-being through their academic achievement. Academic achievement negatively affects adolescents’ subjective well-being. The research results strongly support the correctness of the theoretical framework, indicating the complexity of the formation of adolescents’ subjective well-being.

## Introduction

The term Subjective well-being (SWB) was first introduced by Diener (1984) as a tool to identify psychological states and try to understand people’s evaluations of their quality of life, including both their cognitive judgments and affective reactions (Diener et al., 2018). Subjective well-being is the individual’s perception and experience of positive and negative emotional responses, as well as global and (domain) specific cognitive evaluations of life satisfaction. It is defined as “a person’s cognitive and affective evaluation of his or her life” (Suzuki and Urabe, 2020). Subjective well-being has three components: life Satisfaction, positive affect and negative affect. People who are more satisfied with their lives and experience positive affects more often (e.g., happiness and optimism) and negative affects less often (e.g., sadness and anger) tend to have higher subjective well-being. On the contrary, if an individual is dissatisfied with life, experiences little happiness, and frequently feels negative emotions such as anger or anxiety, the subjective well-being of the individual is lower (Diener et al., 2018). Originated from positive psychology, subjective well-being is considered as a positive attitude toward life. In the Chinese context, subjective well-being mainly refers to the overall evaluation of the quality of life made by individuals according to the standards set by themselves, including two basic components, life satisfaction and emotional experience. And studies on subjective well-being indicate that individual factors and environmental factors are the two main factors affecting subjective well-being (Newland et al., 2019; Villani et al., 2019; Paredes et al., 2021). Personality traits and temperament factors (e.g., introversion and extroversion) can largely explain the difference in subjective well-being (Proctor, 2014). A large number of empirical studies have shown that personality is one of the most important factors predicting subjective well-being, and different kinds of personality traits have diverse effects on the cognitive and emotional components of subjective well-being (Lampropoulou, 2018; Anglim et al., 2020). A study by Lampropoulou (2018) on 714 Middle school students in Greece shows that individual personality characteristics can explain part of the differences in subjective well-being, and the two dimensions of neuroticism and agreeableness can significantly predict subjective well-being. Busseri (2018)focus on optimism and its relationship to subjective well-being. The best fitting model suggests that the direct effect of optimism on global life satisfaction is stronger than that *via* affectivity.

As for environmental factors, numerous studies have shown that family and school environment play a vital role in shaping adolescents’ subjective well-being (Lee and Yoo, 2015; Lampropoulou, 2018; Newland et al., 2019). Different types of family capital, including economic, social and cultural capital, have an important impact on the development of subjective well-being of adolescents (Coleman, 1988; Bradley and Corwyn, 2002; Magnuson, 2007; Parcel et al., 2010). Studies on Chinese adolescents also reached similar conclusions (Ge, 2015; Du, 2017). Inequality of resources within a family also extends to other social situations, such as schools and communities, thus affecting the subjective well-being of students (Parcel et al., 2010). Surrounding environments of adolescents, including family, school, community and society, are important factors in predicting adolescents’ subjective well-being.

Interest in exploring and predicting students’ subjective well-being has been growing these years as it is regarded as a prerequisite for improving people’s mental health. For researchers, the issue of how to promote the development of children’s and adolescents’ subjective well-being has drawn great attention (Diener et al., 2018).China has been deeply influenced by Confucian culture since ancient times. As talents are usually selected by examination, Chinese people attach great importance to education. Children’s academic achievement is generally valued by parents, teachers and even the whole society. In fact, under the Policy of Diversion of General Education and Vocational Education in Entrance Examination for Senior Middle School, the pressure of Chinese teenagers to go to school has moved forward to the junior high school stage. Some Chinese students improve their exam results at the cost of happy experience they should have, and their academic life lacks vitality and motivation for sustainable development (Steel et al., 2018). Judging from international investigation, there is indeed a phenomenon of “high achievement but low well-being” in China. According to the PISA 2015 results, Chinese students from four provinces and cities (Beijing, Shanghai, Jiangsu and Guangdong) had an average score of 518 in science literacy, ranking 10th among all participating countries, which was higher than the average performance of OECD countries. Meanwhile, the well-being index of students in China mainland (6.83), Macao (6.59), Taiwan (6.59), and Hong Kong (6.48) was significantly lower than the average level of OECD countries (7.31), indicating that Chinese students have high academic attainment but low well-being. It also raises a question about current Chinese education: do we have to sacrifice student’ well-being in exchange for higher academic achievement? In addition, what impact academic achievement has on adolescents’ subjective well-being, especially for Chinese middle school students who undertake strong academic pressure, is one of the topics that this paper aims to explore. Most studies suggest that adolescents’ academic achievement is positively correlated with their life satisfaction (Bucker et al., 2018; Steinmayr et al., 2018; Suzuki et al., 2019). Studies on Chinese students show similar results, with academic performance considered to be a predictor of individual subjective well-being (Cao and Yuan, 2018). Although some studies present different conclusions (Bucker et al., 2018; Rand et al., 2020), academic achievement is at least partially correlated with the overall subjective well-being, even if they are not completely correlated.

As presented above, the available social resources have a great influence on adolescents’ subjective well-being, among which the resources obtained from their families and schools have the most far-reaching impact. In the Chinese context, family capital can be approximately understood as the socioeconomic background of a family, it can provide a variety of useful resources for individual action. Moreover, there is a close relationship between subjective well-being and academic achievement. However, there are still some deficiencies in the existing studies. On the one hand, subjective well-being is often discussed ignoring different social and cultural background, which may lead to inconsistencies in research findings. Some studies have shown that cultural differences may lead to differences in subjective well-being of adolescents from different countries (Lampropoulou, 2018; Villani et al., 2019; Paredes et al., 2021). In China, adolescents are likely to place a higher value on academic achievement than students in other countries, and families and schools will pay more attention and expectations to their children’s academic achievement. Therefore, care and pressure from family and school will affect the development of Chinese adolescents’ subjective well-being to some extent. On the other hand, though many studies explored the factors that influence subjective well-being, few studies discussed the relationship between these factors. Indeed, inequality in family resources tends to further magnify its role after students enter school, rendering inequality in student outcomes (Lampropoulou, 2018). There are diverse external influencing factors of students’ subjective well-being, whose effect size and influencing paths are quite complex, so further research is necessary.

**Figure 1:**
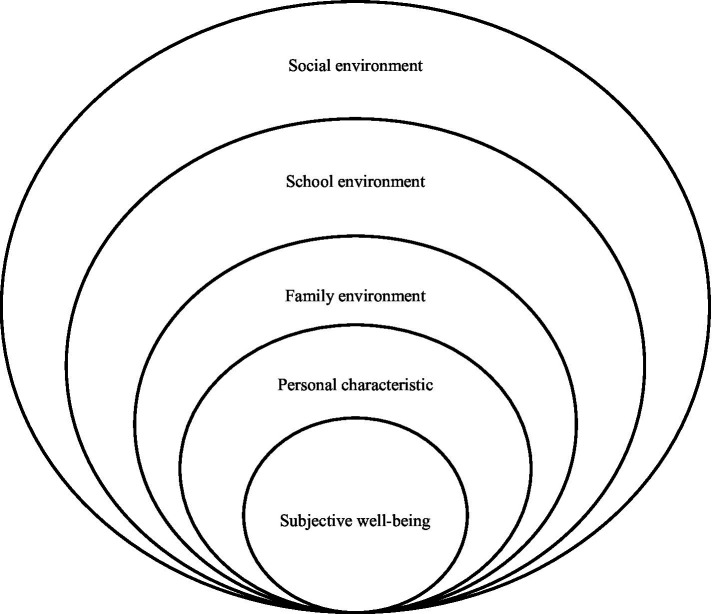
Influencing factors of adolescents’ subjective well-being.

Based on this, this study will explore the influencing factors of subjective well-being from the perspective of Bronfenbrenner’s ecological systems theory, which holds that there are mutual and dynamic influences between individuals and environment. According to the differences in the degree and manner of the system’s influence on people, Bronfenbrenner structured and concreted the system, and established the connection between different systems, which is helpful for the analysis of the influencing factors of a specific problem. In his theory, children typically find themselves enmeshed in five successive ecosystems, namely microsystem, mesosystem, exosystem, macrosystem and chronosystem. According to ecological systems theory, human development refers to “stability and change in the biopsychological characteristics of human beings over the life course and across generations,” which “takes place through processes of progressively more complex reciprocal interaction between an active, evolving biopsychological human organism and the persons, objects, and symbols in its immediate external environment” (Bronfenbrenner and Morris, 2007). In other words, the factors affecting adolescents’ subjective well-being comprise social environment, school environment, family environment and personal characteristics (see Figure 1). In terms of exosystem and macrosystem, they mainly represent the power of social environment and social culture. Under the impact of various environmental forces, Chinese adolescents draw an equal sign between high academic achievement and success, and therefore regard improving academic achievement as the main method to improve their subjective well-being. In addition, considering the uniqueness of China’s current college-oriented background and the indispensable role of individual characteristics in shaping subjective well-being, individual academic achievement, as the external embodiment of individual achievement goals, is an important psychological motivation for middle school students to consistently pursue and obtain subjective well-being. Therefore, this study believes that academic achievement is an important personal characteristic that affects the subjective well-being of adolescents.

Since this study will not focus on social environment, we will explain how family environment represented by family capital, school environment represented by school climate and individual academic achievement affect the adolescents’ subjective well-being. Attempts to establish a theoretical model of family capital influencing the adolescents’ subjective well-being from the perspective of Bronfenbrenner’s ecological systems theory, to explore the relationship between family capital, school climate and students’ academic achievement, and to test their effects on adolescents’ subjective well-being. To be specific, this study believes that family capital is a decisive factor affecting adolescents’ subjective well-being. Family capital will influence adolescents’ academic achievement through school climate, thus affecting adolescents’ subjective well-being.

## Methodology

In view of the research object, research question and the nature of the problem, this paper conducts an empirical research based on the philosophical basis of post-positivism. Firstly, a theoretical model of the influence of family capital on adolescents’ subjective well-being is established, and the research hypothesis of this paper is put forward. Secondly, the role of family capital on adolescents’ subjective well-being and its influencing factors is investigated empirically by using structural equation model.

### Research hypothesis

#### Family capital and access to educational opportunities

According to the interpretation of social capital theory by different scholars, this study divides family capital into family economic capital, family social capital and family cultural capital.

Economic capital refers to the economic resources that can be used collectively by individuals or families, which enhance parents’ ability to provide their children with basic necessities of life. Children without basic necessities of life tend to develop poorly (Huston et al., 1994). Besides, economic capital not only provides food, shelter and clothing, but also provides higher quality communities, better schools and more experiences for cognitive and social development—all of which are associated with more favorable children development outcomes (Cheng and Kaplowitz, 2016; Herrero and Hughes, 2019; Ge, 2020). Families with more financial resources are capable of promoting their children’s educational achievement through paying for tutoring, private school education, and higher college tuition fees, while parents with less financial security may not be able to provide these (Ye et al., 2020).

Social capital refers to the connection between individuals that produces social outcome (Coleman, 1988), which reflects the relationship between people (Bourdieu, 1973). It also shows how individuals can benefit from participating in groups (Bourdieu, 1973; Coleman, 1988). For example, the bond between parents and children and the time and attention parents give to their children promotes their social norms and cognitive development, and ultimately educational achievement (Li et al., 2018; Hunter et al., 2019; Waddling et al., 2019) argues that family social capital contains the quality of the relationship between children and their parents. Parental concern for children, parental supervision, extended family communication and support are also forms of family social capital. Studies have shown that the higher the level of family social capital, the better adolescents’ academic performance (Carolan and Wasserman, 2015; Kalil, 2015; Ren et al., 2021), and the longer years of education (An and Western, 2019).

French scholar Bourdieu (1984) first put forward the concept of cultural capital. In terms of individual inequality in academic achievement, he argues that these inequalities stem from the different appropriation of cultural capital by individuals and families, that is, familiarity with high-status cultural norms, etiquettes and behaviors, and the ability to express that familiarity effortlessly. The more cultural capital parents have, the more they will attach importance to their children’s education. They try to teach them by words and deeds through family cultural atmosphere, so that their children will attach importance to academic achievement, and thus enable their children to receive higher quality education. There are two explanations for how cultural capital promotes academic achievement. The first explanation emphasized by Bourdieu is that cultural capital signals academic talent to teachers, which in turn leads to favoritism, preferential treatment and educational success. The second explanation is that cultural capital can cultivate children’s skills, such as analytical ability and creativity, directly enhancing educational success (Breinholt and Jæger, 2020).

Plenty of studies have supported the link between family capital and individual access to educational opportunities. The research of Reardon (2019) reveals that the role of schools in creating educational opportunities varies with different school districts. Moreover, access to early education opportunities is closely related to the socioeconomic characteristics of the region, that is, rich families and regions can offer much greater opportunities in early childhood than poor families. In research, family cultural capital (represented by parents’ years of schooling) and family social capital (represented by father’s career) significantly affect children’s entrance opportunity for higher education, while family economic capital (represented by annual family income) does not significantly affect children’s access to higher education. Research by Stahl et al. (2018) demonstrates that migrant children, especially those with less educated parents, tends to participate in early childhood education and care centers with lower levels of quality, while children from poor or single-parent families have few obvious disadvantages. In conclusion, though it still remains vague that what specific type of family capital plays a greater role in influencing access to school education, family capital can positively influence adolescents’ access to school education. As little research discusses whether family capital influences school climate, given that rich family capital tends to provide adolescents with better school education opportunities, it is reasonable to assume that adolescents with affluent family capital will assemble in better schools, thus creating better school climate, which is consistent with Putnam’s study (Putnam, 2016). Children from affluent families create a positive learning environment in schools, while children from slums create violence and chaos that ultimately affects everyone in the school.

#### School climate and student outcomes

School climate has been described as “the quality and character of school life” (Cohen et al., 2009), “heart and soul of the school” (Freiberg and Stein, 1999) and “the quality of relationships among students, teachers and school staff” (Konold et al., 2018). While opinions vary on the definition of school climate, most scholars agree that school climate is a multi-dimensional construct, which represents “almost every aspect of school experience” (Wang and Degol, 2016; Bear et al., 2018). Although researchers have not yet reached a consensus on the indicators that constitute school climate, four spheres of school climate have emerged in previous studies, which are safety, teaching and learning, school community, and institutional environment (Thapa et al., 2013; Wang and Degol, 2016; Eichert et al., 2018). Safety includes maladaptive behaviors such as bullying, classroom discipline problems, substance abuse and absenteeism, as well as rules, attitudes and school strategies related to these maladaptive behaviors. Teaching and learning focuses on teaching aspects, such as academic support, teacher training, curricula and professional development. School community emphasizes the quality of interpersonal relationships within the school. Institutional environment reflects school resources and indicators of school organization.

Student academic achievement, often referred to as academic achievement, has long been seen as the result of a changing school climate. The quality of school academic environment, as an important indicator of student achievement, has been widely demonstrated in the sample of primary, middle and high school students (La Salle et al., 2018; Yang et al., 2018; Karpudewan, 2019) Characterized by effective leadership by teachers and principals who believe they have the ability to improve student performance, high-achieving schools tend to emphasize the importance of a commitment to high academic standards. Ma and Wilkins (2002) find that schools with greater academic pressure, i.e., schools that maintain high standards and encourage students to do their best—have a stronger growth-promoting effect on students’ science achievement. Some studies suggest that a school’s achievement goal structure may directly or indirectly affect students’ achievement through their motivational beliefs (Koch, 2018; Steinmayr et al., 2018).

Some studies verify how school climate is associated with students’ psychological and social–emotional functioning, including psychopathological problems such as depression and anxiety, as well as positive adjustment (e.g., high self-esteem, adaptive coping strategies, and high life satisfaction) (Durlak et al., 2011; Weeldenburg et al., 2020). In addition to alleviating and regulating students’ psychological problems, school climate is also conducive to improving students’ satisfaction and well-being. For instance, Zullig et al. (2011) illustrate that school climate is correlated with school satisfaction in five domains, which are academic support, positive teacher-student relationship, school connection, order and discipline, and academic satisfaction, respectively. Lombardi et al. (2019) finds that school climate is an important factor to improve students’ participation in school activities, but it is effective only if it improves students’ experience of well-being. Additionally, promoting school climate means direct support for good well-being experiences and indirect support for students’ participation in school activities, regardless of individual learning ability and personality characteristics.

In summary, good school climate is not only beneficial to promoting students’ academic performance and preventing disruptive behavior, and, more importantly, it can effectively improve students psychological experience. Students will keep in a good mood within good school climate, which eventually helps improve students’ life satisfaction and subjective well-being.

#### Academic achievement and subjective well-being

Academic achievement and subjective well-being are both playing important roles in adolescents’ daily life. Most studies show a significant correlation between them. Bucker et al. (2018) investigated whether self-regulation, well-being and exercise behavior play a crucial role in influencing the academic performance of Swedish high school students. The result of the questionnaire shows that students’ GPA is positively correlated with subjective well-being and psychological well-being. This research supports that there is a positive correlation between adolescents’ subjective well-being and their academic performance, and also indicates that students can improve their well-being level by adjusting self-regulation strategies, so as to improve their academic performance. Research by Karvonen et al. (2018) shows that, students’ family background is associated with differences in well-being and academic achievement at the school level. Although some studies demonstrate that adolescents’ academic achievement is not related to subjective well-being (Steinmayr et al., 2016; Rand et al., 2020), most literature supports the positive correlation between academic achievement and subjective well-being.

Studies have supported that students’ academic achievement is one of the factors that predict adolescents’ subjective well-being. For instance, Asadullah et al.’s (2018) study shows that both male and female adolescents’ academic performance can positively predict positive affect, and valence toward school can moderate the influence of academic performance on positive affect in male groups. Cao and Yuan (2018) explore the relationship between subjective well-being of and academic performance, academic self-concept, and views of happiness of primary and secondary students. The results of regression analysis illustrate that subjective well-being in grade 6 can significantly predict subjective well-being in grade 7, 8, and 9. Meanwhile, academic achievement at the end of fifth grade is a significant predictor of seventh—and ninth-grade subjective well-being. In contrast, there are also studies supporting that the combination of adolescents’ positive cognition, emotion and relationship constitute subjective well-being, and thus generate educational benefits (Samuel et al., 2013; Putwain et al., 2019). However, it is not clear whether academic achievement promotes subjective well-being or subjective well-being enhances academic achievement. As pointed out by Steinmayr et al. (2016), we can assume that academic achievement predicts changes in students’ subjective well-being and that subjective well-being predicts changes in academic achievement.

#### The influence of family capital on adolescents’ academic achievement

As a vital part of students’ development outcomes, researchers often associate adolescents’ academic achievements with family capital. Numerous studies have shown that family capital has a positive impact on students’ academic achievements (Furstenberg and Hughes, 1995; Cheng and Kaplowitz, 2016; Herrero and Hughes, 2019; Ge, 2020). Researchers pay particular attention to the positive effects of family economic capital and family social capital on adolescents. For example, Letourneau et al. (2013) explore the relationship between parents’ socioeconomic status and children’s cognitive development through meta-analysis. The higher the income to needs ratio or the greater the family wealth, the higher the children’s cognitive test scores. In addition, the development of children’s language and literacy skills is related to parents’ socioeconomic status. It is worth mentioning that the results of several studies indicate that the relationship between socioeconomic status and children’s development is mediated by parent–child relationship. It is not difficult to find that students’ academic achievement is not only closely related to the family economic capital, but also mediated and influenced by family social capital.

Moreover, family cultural capital also positively affects adolescents’ academic achievement. Crede et al. (2015) explore the potential moderating effect of parental education on academic achievement and life satisfaction among German adolescents. The results imply that mother’s education level plays a moderating role in the relationship between academic achievement and student life satisfaction, proving that mother’s education level can predict the children’s academic achievement. Breinholt and Jæger (2020) analyze the relationship between cultural capital and educational performance with the data of ECLS-K and ECLS-K:2011 in the United States. The empirical results show that cultural capital works through signals of skill rather than academic ability. One aspect of cultural capital, namely children’s interest in reading, has a direct positive impact on reading and math test scores, suggesting that family’s literary environment can improve children’s educational performance *via* developing children’s skills, like complex vocabulary, creativity and cultural knowledge.

#### Family capital and subjective well-being

Parents are active and important agents who invest family capital in their children, with the expectations that these investments will pay off in terms of future child and adolescent well-being (Parcel and Hendrix, 2014). Children’s well-being is one of the expected returns from parents’ investment in family capital, which attracted the attention of many researchers. Most studies support the positive effect of family capital on student subjective well-being. For example (Asadullah et al., 2018; Casas and Gonzalez-Carrasco, 2019), have shown that, at the macro level, personal well-being (i.e., life satisfaction) is associated with poverty, housing conditions and relationships with classmates (as well as age and gender). At the micro level, British children’s personal well-being is only weakly correlated with age, gender and poverty. However, unlike at the macro level, personal well-being is also associated with family structure, school and neighborhood well-being, and is more strongly correlated with family well-being (parent–child relationship). Steinmayr et al. (2018) on sixth grade students in Shenzhen reveals that, in addition to parents’ human capital and financial capital, family social capital and school social capital explain a large number of differences in the subjective well-being of sixth grade students. Specifically, children who think they have a close relationship with their parents, teachers and peers are less likely to feel negative subjective well-being. However, other studies have questioned the strength family capital’s impact on adolescents’ subjective well-being. An analysis of more than 13,000 children in United Kingdom by Rees (2018) found that family and socioeconomic variables measured at 9 months of age, such as household income and family structure, explained only a small fraction of the change in subjective well-being at the age of 11.

In general, family capital can promote adolescents’ subjective well-being. Although some literature questioned the strength of the relationship between them, it is universally accepted that family capital has a positive effect on adolescents’ subjective well-being. Even if family capital is not related to subjective well-being at all domains, it is at least more or less related to different dimensions of subjective well-being. Longitudinal studies on subjective well-being do not fully account for the impact of family capital, but the results of cross-sectional studies are mostly significant.

**Figure 2:**
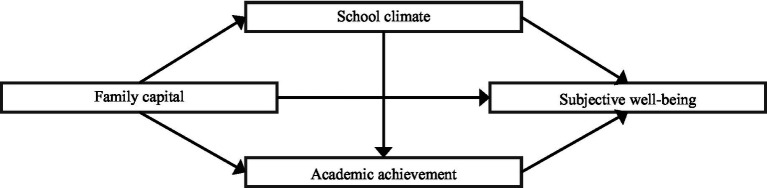
Theoretical model of the influence of family capital on adolescents’ subjective well-being.

Based on the above theoretical analysis, this paper propose a theoretical model of the influence of family capital on the subjective well-being of adolescents, as presented in Figure 2.

Further, this paper put forward the following assumptions:

*H1*: Adolescents’ family capital has a significant positive influence on school climate.

*H2*: School climate has a significant positive influence on adolescents’ academic achievement.

*H3*: School climate has a significant positive influence on adolescents’ subjective well-being.

*H4*: Adolescents’ academic achievement has a significant positive impact on their subjective well-being.

*H5*: Adolescents’ family capital has a significant positive influence on their academic achievement through school climate.

*H6*: Adolescents’ family capital has a significant positive effect on their subjective well-being through school climate.

### Research design

#### Data collection method

The data used in this study are from the public test data of PISA (2018) (As of the time of finalization of the paper, PISA 2021 data has not been released). PISA is the OECD’s program for international student assessment, a triennial assessment that measures the ability of 15-year-olds around the world to apply their knowledge and skills in reading, mathematics and science to meet real-world challenges. In addition to its focus on reading, math and science literacy, PISA measures students’ overall qualities, including cooperative problem solving, financial literacy and global competency, as well as their developmental status, including students’ well-being. Guided by modern educational measurement theories such as item response theory, PISA survey questionnaire is designed by international education experts and measurement experts in cooperation, whose results are obtained *via* professional statistical software. The data of PISA has the following three characteristics: the content of the evaluation is focused, the evaluation methods are diverse and scientific, the evaluation results can provide a basis for educational decision-making.

Data from four Chinese provinces and cities in the PISA (2018) database are selected as research samples. Four Chinese mainland provinces and municipalities participated in PISA (2018) test, namely Beijing, Shanghai, Jiangsu and Zhejiang. A total of 12,058 students from 361 schools in four provinces and cities participated in the test on behalf of students from these four provinces and cities. Data used in this study is only extracted from student questionnaire. After deleting the missing values, the results of 10,805 middle school students are used, and the specific distribution of the participants is shown in Table 1.

**Table 1:** Distribution of participants (*n* = 10,805).

	*N*	(%)
*Gender*		
Female	5,249	48.6
Male	5,556	51.4
		
*Type of school*		
Junior high school	3,793	35.1
Senior high school	5,176	47.9
Vocational junior secondary school	9	0.1
Secondary Vocational School, 5-years vocational school	1,827	16.9
		
*Grade*		
7th	22	0.2
8th	153	1.4
9th	3,627	33.6
10th	6,878	63.7
11th	118	1.1
12th	7	0.1

#### Variables description

The variables used in this study are directly or indirectly derived from student questionnaire of PISA (2018). The dependent variable of this study is adolescents’ subjective well-being, while the independent variable includes factors of family capital, school climate and academic achievement. This section will make a brief explanation on these variables, and more details are presented in the appendix.

##### Subjective well-being

Adolescents’ subjective well-being is the key variable of this study. It is generally believed that subjective well-being has two dimensions: cognitive dimension, which generally refers to people’s overall life satisfaction, and emotional dimension, which can be divided into the measurement of people’s positive affect and negative affect. In terms of the cognitive dimension of adolescents’ subjective well-being, this study adopts item ST016 in the student questionnaire for measurement. As for the emotional dimension, item ST186 investigates the frequency of positive and negative emotions that students often feel. In order to better distinguish adolescents’ positive and negative affect, it is necessary to carry out factor analysis on this question. After KMO and Bartlett’s Test, this item is suitable for factor analysis. Rotated component matrix is shown in Table 2. As can be seen from Table 2, factor F1 has high loadings on happy, lively, proud, joyful and cheerful, which can be named as positive affect factor. Factor F2 has high loadings on scared, miserable, afraid and sad, which should be called negative affect factor. The calculated positive affect and negative affect factor scores are stored as new variables for later analysis.

**Table 2:** Rotated component matrix of ST186.

	F1	F2
Happy	0.804	
Scared		0.773
Lively	0.726	
Miserable		0.711
Proud	0.569	
Afraid		0.788
Joyful	0.825	
Sad		0.744
Cheerful	0.791	

##### Family capital

The measurement of latent variable of family capital is achieved through the measurement of family economic capital, family social capital and family cultural capital. PISA (2018) does not directly ask adolescents about personal family wealth and socioeconomic status, but asks several different questions to understand the general status of family assets and parents’ socioeconomic status, and then calculates related derived variables through item response theory. To be specific, the measurement of family economic capital and family cultural capital is achieved by calculating three variables, including WEALTH (Family wealth), CULTPOSS (Cultural possessions at home) and HERDES (Home educational resources). Family social capital is measured by Index highest parental education in years of schooling (PARED) and Index highest parental occupational status (HISEI).

##### School climate

In general, school climate contains four dimensions: safety, teaching and learning, school community and institutional environment. These four dimensions comprise almost all the characteristics of school climate, which, taken together, have a lasting impact on students’ academic, behavioral and psychological outcomes. Since this research focuses on the impact of external environment on adolescents’ academic achievements and subjective well-being, according to Wang and Degol (2016), institutional environment has inconsistent effects on students’ academic achievements in different empirical studies, and studies that connected institutional characteristics with psychosocial functioning are scarce. Therefore, this study constructs school climate from three dimensions: safety, teaching and learning, and school community.

First, safety. Safety dimension refers to the physical and social–emotional security, disciplinary environment and the frequency of students’ destructive behaviors of school members. In this study, three continuous variables are used to reflect safety dimension: Student’s experience of being bullied (BEINGBULLIED), Disciplinary climate in Chinese lessons (DISCLIMA) and destructive behaviors. Among them, disruptive behaviors refer to student truancy and lateness. In PISA (2018), students were asked to answer how often they had skipped a whole school day, skipped some classes and arrived late for school in the last 2 weeks of school in item ST062. Based on the students’ answers to this question, this study constructed the variable of destructive behaviors through principal component analysis, whose composition matrix is shown in Table 3. We store the extracted common factor score as a new variable and name it as disruptive behaviors.

**Table 3:** Component matrix of ST062.

	F1
Skipped a whole school day	0.813
Skipped some classes	0.860
Arrived late for school	0.717

Second, teaching and learning. Teaching and learning dimension refers to classroom practice and teacher behavior that shape learning experience and promote adolescents’ social–emotional development. In this study, five variables are used to measure the quality of teaching and learning, which are TEACHINT (Perceived teacher’s interest), DIRINS (Teacher-directed instruction), PERFEED (Perceived feedback), TEACHSUP (Teacher support in Chinese lessons), and ADAPTIVITY (Adaptation of instruction).

Third, school community. School community dimension refers to the community relationship established by students, teachers, principals, parents and local communities within the school setting. This study measures school community dimension from three aspects: Sense of belonging to school (BELONG), Perception of cooperation at school (PERCOOP) and Parents’ emotional support perceived by student (EMOSUPS).

##### Academic achievement

In this study, adolescents’ academic achievement is reflected by their subject literacy scores in PISA (2018). Subject literacy refers to a student’s ability to apply knowledge and skills in key areas and to analyze, reason and communicate effectively in identifying, explaining and solving problems in a variety of situations. Each round of PISA tests students’ science, mathematics and reading literacy, with one chosen as a major area of assessment. The major domain of PISA (2018) is reading literacy (OECD, 2019). It is worth noting that literacy scores are presented as Plausible values. Plausible value is used to estimate scores for groups of students with similar response patterns and background characteristics. In essence, it is not a kind of capability estimation, but a method and technology (Gao, 2011). As PISA 2015’s technical report points out, plausible value is not a substitute for an individual’s test score. When the theoretical model is correct, plausible values will provide consistent estimates of population characteristics, even if they are not usually unbiased estimates of the capabilities of the individuals (OECD, 2017). As Büyükkıdık et al. (2018) do in their research, this paper takes the arithmetic mean of 10 PV values provided by PISA (2018) raw data as student’s subject literacy score. Table 4 shows all variables included in this study.

**Table 4:** List of variables.

	Code	Cronbach’s alpha
*Subjective well-being*		
Overall life satisfaction	ST016Q01NA	
Positive affect		0.84
Negative affect		0.78
		
*Family capital*		
Cultural possessions at home	CULTPOSS	0.62
Home educational resources	HEDRES	0.55
Family wealth	WEALTH	0.64
Index highest parental education in years of schooling	HISEI	
Index highest parental occupational status	PARED	0.71
		
*Safety*		
Disciplinary climate in Chinese lessons	DISCLIMA	0.89
Student’s experience of being bullied	BEINGBULLIED	0.74
Destructive behaviors		0.44
		
*Teaching and learning*		
Perceived teacher’s interest	TEACHINT	0.89
Teacher-directed instruction	DIRINS	0.81
Perceived feedback	PERFEED	0.89
Teacher support in Chinese lessons	TEACHSUP	0.85
Adaptation of instruction	ADAPTIVITY	0.82
		
*School community*		
Perception of cooperation at school	PERCOOP	0.92
Sense of belonging to school	BELONG	0.82
Parents’ emotional support perceived by student	EMOSUPS	0.91
		
*Academic achievement*		
Mathematics literacy	PVMATH	0.84
Reading literacy	PVREAD	0.91
Science literacy	PVSCIE	0.87

Note: 1. There is only one item of overall life satisfaction, and the variable of Index highest parental education in years of schooling is converted from open-ended questions, so their internal consistency coefficients cannot be calculated; 2. The internal consistency coefficients of DISCLIMA, BEINGBULLIED, TEACHINT, PERFEED, TEACHSUP, PERCOOP, BELONG, PVMATH, PVREAD and PVSCIE are given by PISA’s report, while the internal consistency coefficients of the rest variables are calculated by the author.

#### Data analysis method

The data analysis of this study is divided into two parts. First, SPSS 24 is used to carry out descriptive statistical analysis on student variables, aiming to explain the distribution of samples and the differences between different groups. Next, combining with the theoretical model of the influence of family capital on adolescents’ subjective well-being, AMOS 24 is used to establish the structural equation model, in order to test the research hypothesis proposed previously.

## Results and discussion

### Descriptive analysis

Table 5 presents the means and standard deviations of all variables in the model. It can be seen from this table:

1. In terms of family capital, there is little difference between Chinese students’ Cultural possessions at home, Home educational resources and Family wealth and the average level of OECD. Chinese students’ Cultural possessions at home and Family wealth are slightly lower than the average level of OECD, while their Home educational resources are slightly higher. From the perspective of Chinese students’ family social capital, the Index highest parental occupational status is 53.35, and the Index highest parental education in years of schooling is 12.88, reflecting that Chinese students’ parents are well educated.
2. Among all dimensions of school climate, the mean value of Student’s experience of being bullied in the dimension of safety is negative, indicating that the frequency of Chinese students being bullied in school is slightly lower than the OECD average. The mean value of Disciplinary climate in Chinese lessons is positive, indicating that the Chinese students share better disciplinary climate in language classes than the OECD average. Moreover, the mean value of all variables of Teaching and learning dimension is positive, demonstrating that Chinese students feel higher quality of teachers’ instruction than the average level of OECD on the whole. Finally, in terms of school community, the mean value of Sense of belonging to school is negative, while the mean values of Perception of cooperation at school and Parents’ emotional support perceived by student are positive.
3. In all dimensions of academic achievement, Chinese students’ math literacy is 598.25, reading literacy is 566.40, and scientific literacy is 599.15, all of which are at the international leading level and are consistent with the official results.
4. Among all dimensions of Subjective well-being, the overall life satisfaction of Chinese students (out of 10) is 6.84, lower than the OECD average of 7.04. Besides, Positive affect and Negative affect experienced by Chinese students are higher than the average of all participating countries, indicating that Chinese students experience both positive and negative affect more frequently at school than the average level.

**Table 5:** Descriptive statistics of subjective well-being and its influencing factors.

	M	SD
*Family capital*		
Cultural possessions at home	−0.07	1.14
Home educational resources	0.31	0.98
Family wealth	−0.65	0.81
Index highest parental occupational status	53.35	21.42
Index highest parental education in years of schooling	12.88	3.36
*Safety*		
Disciplinary climate in Chinese lessons	0.85	1.01
Student’s experience of being bullied	−0.25	0.85
Destructive behaviors	−0.59	0.42
*Teaching and learning*		
Perceived teacher’s interest	0.40	0.96
Teacher-directed instruction	0.52	1.01
Perceived feedback	0.43	0.86
Teacher support in Chinese lessons	0.36	1.03
Adaptation of instruction	0.44	1.03
*School community*		
Perception of cooperation at school	0.26	0.99
Sense of belonging to school	−0.12	0.90
Parents’ emotional support perceived by student	0.03	0.92
*Academic achievement*		
Mathematics literacy	598.25	74.04
Reading literacy	566.40	83.37
Science literacy	599.15	77.57
*Subjective well-being*		
Overall life satisfaction	6.84	2.29
Positive affect	0.19	0.85
Negative affect	0.69	0.82

### Correlation analysis

Based on the theoretical model of the influence of family capital on adolescents’ subjective well-being, this study explores the correlation between subjective well-being and its influencing factors through correlation analysis (as is shown in Table 6).

1. Family capital and school climate. Variables of family capital are significantly positively correlated with variables of school climate other than bullying experience and destructive behaviors. Student’s experience of being bullied is significantly negatively correlated with family capital variables other than the highest occupational status of parents, while Destructive behaviors is significantly negatively correlated with family cultural assets and family educational assets.
2. Family capital and academic achievement. In terms of correlation coefficients, all types of family capital were significantly positively correlated with students’ math, reading, and science literacy, with correlation coefficients ranging from 0.18 to 0.33.
3. School climate and academic achievement. In general, while Teacher support in Chinese lessons are not significantly correlated with any type of academic achievement, Student’s experience of being bullied, Destructive behaviors and Teacher-directed instruction are significantly negatively correlated with academic achievement, and Disciplinary climate in Chinese lessons, Perceived teacher’s interest, Perceived feedback, Adaptation of instruction, Perception of cooperation at school, Sense of belonging to school and Parents’ emotional support perceived by student are significantly positively correlated with academic literacy of all subjects.
4. Subjective well-being and its influencing factors. First, among all variables of family capital, except that Index highest parental education in years of schooling have nothing to do with adolescents’ subjective well-being, family capital is significantly positively correlated with adolescents’ subjective well-being in general, both in the emotional dimension and the cognitive dimension. Second, in terms of school climate, all variables except Student’s experience of being bullied and Destructive behaviors are positively correlated with adolescents’ overall life satisfaction and positive affect, and are negatively correlated with negative affect, while Student’s experience of being bullied and Destructive behaviors are opposite. Third, academic achievement of all subjects is negatively correlated with students’ overall life satisfaction, but not significantly correlated with students’ positive affect. It is worth noting that reading literacy and science literacy scores are significantly positively correlated with students’ negative affect.

**Table 6:** Correlation matrix of subjective well-being and its influencing factors.

	1	2	3	4	5	6	7	8	9	10	11	12	13	14	15	16	17	18	19	20	21	22
1	1																					
2	0.46^*^	1																				
3	0.47^*^	0.42^*^	1																			
4	0.37^*^	0.26^*^	0.43^*^	1																		
5	0.38^*^	0.30^*^	0.42^*^	0.60^*^	1																	
6	0.15^*^	0.18^*^	0.11^*^	0.10^*^	0.13^*^	1																
7	−0.05^*^	−0.11^*^	−0.05^*^	−0.02	−0.03^*^	−0.23^*^	1															
8	−0.03^*^	−0.05^*^	0.00	−0.02	−0.02	−0.17^*^	0.15^*^	1														
9	0.15^*^	0.18^*^	0.11^*^	0.10^*^	0.13^*^	0.38^*^	−0.17^*^	−0.13^*^	1													
10	0.10^*^	0.14^*^	0.06^*^	−0.01	0.00	0.26^*^	−0.13^*^	−0.06^*^	0.44^*^	1												
11	0.10^*^	0.14^*^	0.07^*^	0.03^*^	0.05^*^	0.27^*^	−0.17^*^	−0.09^*^	0.47^*^	0.60^*^	1											
12	0.13^*^	0.16^*^	0.12^*^	0.06^*^	0.10^*^	0.27^*^	−0.08^*^	−0.05^*^	0.54^*^	0.48^*^	0.42^*^	1										
13	0.13^*^	0.16^*^	0.08^*^	0.05^*^	0.07^*^	0.30^*^	−0.12^*^	−0.10^*^	0.53^*^	0.48^*^	0.48^*^	0.57^*^	1									
14	0.12^*^	0.16^*^	0.09^*^	0.07^*^	0.08^*^	0.23^*^	−0.18^*^	−0.08^*^	0.36^*^	0.27^*^	0.27^*^	0.31^*^	0.30^*^	1								
15	0.14^*^	0.17^*^	0.13^*^	0.09^*^	0.10^*^	0.22^*^	−0.27^*^	−0.09^*^	0.31^*^	0.23^*^	0.24^*^	0.26^*^	0.24^*^	0.37^*^	1							
16	0.18^*^	0.21^*^	0.14^*^	0.11^*^	0.13^*^	0.16^*^	−0.12^*^	−0.09^*^	0.30^*^	0.19^*^	0.21^*^	0.22^*^	0.22^*^	0.29^*^	0.31^*^	1						
17	0.23^*^	0.22^*^	0.18^*^	0.31^*^	0.31^*^	0.20^*^	−0.03^*^	−0.13^*^	0.13^*^	−0.07^*^	0.03^*^	0.01	0.09^*^	0.08^*^	0.07^*^	0.18^*^	1					
18	0.27^*^	0.25^*^	0.20^*^	0.33^*^	0.33^*^	0.21^*^	−0.05^*^	−0.15^*^	0.13^*^	−0.06^*^	0.03^*^	0.01	0.08^*^	0.05^*^	0.06^*^	0.17^*^	0.89^*^	1				
19	0.25^*^	0.24^*^	0.20^*^	0.32^*^	0.31^*^	0.20^*^	−0.01	−0.15^*^	0.12^*^	−0.08^*^	0.02	0.01	0.07^*^	0.06^*^	0.07^*^	0.14^*^	0.92^*^	0.94^*^	1			
20	0.05^*^	0.12^*^	0.06^*^	0.01	0.02	0.18^*^	−0.21^*^	−0.07^*^	0.27^*^	0.23^*^	0.22^*^	0.23^*^	0.19^*^	0.29^*^	0.37^*^	0.26^*^	−0.04^*^	−0.07^*^	−0.05^*^	1		
21	0.10^*^	0.14^*^	0.07^*^	0.01	−0.01	0.11^*^	−0.17^*^	−0.04^*^	0.21^*^	0.17^*^	0.18^*^	0.18^*^	0.16^*^	0.27^*^	0.38^*^	0.25^*^	0.01	0.01	0.00	0.38^*^	1	
22	0.04^*^	−0.01	0.01	0.03^*^	0.01	−0.12^*^	0.21^*^	0.05^*^	−0.11^*^	−0.10^*^	−0.11^*^	−0.09^*^	−0.09^*^	−0.13^*^	−0.26^*^	−0.1^*^	0.00	0.06^*^	0.03^*^	−0.30^*^	0.01	1

* *p* < 0.001.

1. Cultural possessions at home, 2. Home educational resources, 3. Family wealth, 4. Index highest parental education in years of schooling, 5. Index highest parental occupational status, 6. Disciplinary climate in Chinese lessons, 7. Student’s experience of being bullied, 8. Destructive behaviors, 9. Perceived teacher’s interest, 10. Teacher-directed instruction, 11. Perceived feedback, 12. Teacher support in Chinese lessons, 13. Adaptation of instruction, 14. Perception of cooperation at school, 15. Sense of belonging to school, 16. Parents’ emotional support perceived by student, 17. Mathematics literacy, 18. Reading literacy, 19. Science literacy, 20. Overall life satisfaction, 21. Positive affect, 22. Negative affect.

From the perspective of correlation analysis, it can be seen that multiple dimensions of family capital, school climate and academic achievement are significantly correlated with adolescents’ subjective well-being, but the specific impact path and effect size of each variable should be further analyzed.

Overall life satisfaction, 21. Positive affect, 22. Negative affect.

### Path analysis

In order to further understand the possible interaction between adolescents’ subjective well-being and its influencing factors, this study uses structural equation model to further analyze the influence of family capital, school climate and academic achievement on adolescents’ subjective well-being. Goodness of fit is shown in Table 7, indicating that the theoretical model conforms to the data well as indexes except CMIN/DF all exceed the critical value.

**Table 7:** Goodness of fit.

	CMIN	DF	CMIN/DF	RMSEA	NFI	RFI	IFI	CFI	GFI	AGFI
Result	5586.31	198	28.214	0.05	0.944	0.934	0.946	0.946	0.954	0.942
Standard			< 5.0	< 0.10	> 0.90	> 0.80	> 0.90	> 0.90	> 0.90	> 0.80

**Figure 3:**
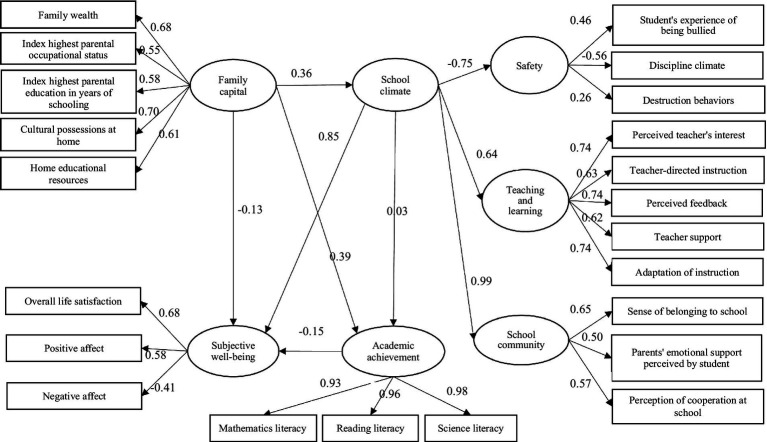
The path model of family capital influencing adolescents’ subjective well-being.

All the path effects in the model are significant(as presented in Figure 3). According to path model, family capital directly has a significant negative impact on adolescent’ subjective well-being, but also plays a mediating role in indirectly influencing adolescent’ subjective well-being through school climate and academic achievement. To be specific, family capital has an indirect positive impact on adolescent’ subjective well-being through school climate, and has an indirect negative impact on adolescent’ subjective well-being through academic achievement. Firstly, academic achievement is found to be directly influenced by family capital and school climate. Moreover, we find that family capital and school climate are positively associated. Thus, hypothesis H1, H2 and H5 have been supported. This indicates that family capital can not only directly and positively affect Chinese adolescents’ academic achievement, but also can play an indirect role by promoting school climate. The path coefficient of direct effect of family capital on academic achievement is 0.39, and the indirect effect of family capital on academic achievement through school climate is 0.0108, indicating that family capital has a stronger direct effect on academic achievement.

Secondly, although school climate can positively affect subjective well-being, contrary to our expectations, academic achievement has a negative effect on subjective well-being. Hypothesis H3 has been verified, while H4 has not. This may suggest that for Chinese students, positive school climate is conducive to the improvement of their subjective well-being, but the improvement of academic performance is not, which shows that Chinese students are under greater academic pressure from a sideways perspective.

Finally, though the path coefficient of family capital on subjective well-being is negative (−0.13), and its indirect effect through academic achievement is also negative (−0.1392). However, family capital’s effect through school climate is strong and positive (0.306), which results in a positive overall effect of family capital on subjective well-being (as is shown in Table 8). Therefore, hypothesis H6 has been verified. Since the total influence of family capital on the adolescents’ subjective well-being is positive, it demonstrates that the positive influence of family capital through school climate is stronger than its negatively direct influence through academic achievement. This suggests that good school climate helps offset the loss of subjective well-being that students experience in the process of improving their academic achievement.

**Table 8:** Standardized effects on adolescent’ subjective well-being.

	Direct effects	Indirect effects	Total effects
Family capital	−0.128	0.249	0.121
School climate	0.853	−0.05	0.848
Academic achievement	−0.147		−0.147

And the summary research hypothesis test results are shown in Table 9.

**Table 9:** Summary of test results for research hypotheses.

**Serial number**	**Hypothetical content**	**Test result**
H1	Adolescents’ family capital has a significant positive influence on school climate	Confirmed
H2	School climate has a significant positive influence on adolescents’ academic achievement	Confirmed
H3	School climate has a significant positive influence on adolescents’ subjective well-being	Confirmed
H4	Adolescents’ academic achievement has a significant positive impact on their subjective well-being	Not confirmed
H5	Adolescents’ family capital has a significant positive influence on their academic achievement through school climate	Confirmed
H6	Adolescents’ family capital has a significant positive effect on their subjective well-being through school climate	Confirmed

### Discussion

This study mainly discusses how family capital, school climate and academic achievement collectively influence adolescents’ subjective well-being. The structural equation model constructed on this basis has a good fitting effect, and all the path coefficients are significant, indicating that family capital is the core factor affecting adolescents’ subjective well-being. Moreover, family capital plays its role through school climate and academic achievement. The correctness of the theoretical framework also suggests to some extent that the unique role of academic achievement should be taken into account when discussing adolescents’ subjective well-being, at least when focusing on Chinese adolescents.

School climate has both direct and indirect effects on adolescents’ subjective well-being through affecting academic achievements, and academic achievement has a direct impact on adolescents’ subjective well-being. Hence, it is reasonable to believe that adolescents with richer family capital can enjoy better school education, so that they can work together to create healthy and positive school climate, which further promotes their academic achievement and subjective well-being. The conclusion also reflects the distinguishing function of family capital to some extent. As a field, school has the function of class division and plays a role of widening class gap. By sending their children to different schools, parents can turn their family capital into their children’s academic achievement and other advantages, so as to realize the distinction between different classes and finally achieve the purpose of class reproduction.

It can be seen from the results of this study that although the comprehensive effect of family capital on adolescents’ subjective well-being is positive, the direct effect of family capital on subjective well-being is negative, and its indirect effect through academic achievement is also negative. The results of this study may suggest that parents’ investment in students’ family capital does not always lead to their children’s well-being. This is probably because parental family capital investment aimed at improving children’s academic achievement increases the level of some psychological factors such as academic pressure and test anxiety, thus leading to the decline of subjective well-being. According to Bronfenbrenner’s ecological systems theory, microsystems have the greatest impact on individuals. It is not difficult to imagine that good interpersonal relationship with peers can make adolescents feel pleasant and happy, and teachers’ greetings and diligent instructions can make them feel love and care. Moreover, a secure school can, to a great extent, prevent students from suffering malice from the outside world. Therefore, shaping good school climate is not only conducive to the improvement of students’ academic performance, but more importantly, it can greatly enhance adolescents’ psychological experience so that they can enjoy a positive and happy school life.

## Conclusion, limitations and prospects

This paper has established the theoretical model of the influence of family capital on adolescents’ subjective well-being, and examined the role between adolescents’ subjective well-being and its influencing factors through structural equation model. The following conclusions can be drawn from this study.

Adolescents’ subjective well-being can be derived from factors such as family capital, school climate and academic achievement, by which the theoretical model of the influence of family capital on adolescents’ subjective well-being has been supported. Adolescents’ subjective well-being is both derived from direct and indirect effects. To be specific, family capital has a direct impact on adolescents’ subjective well-being, but also has an indirect impact through school climate and academic achievements. From the perspective of the direction of these influencing factors, positive and negative influencing factors exist simultaneously for adolescents’ subjective well-being, indicating the complexity of the formation of adolescents’ subjective well-being. In terms of the effect size of different factors, family capital has the greatest positive effect on adolescents’ subjective well-being through school climate. On contrary, the direct influence of family capital, the indirect influence of school climate through academic achievement and the direct influence of academic achievement on adolescents’ subjective well-being have relatively slighter but negative effects.

This study also has some limitations. First, as a result of the incomplete data coverage, the conclusions of this study can only represent the situation of a few provinces in China, and do not reflect the overall level of China and the differences between China and other countries. Second, due to the fitting effect of the model, demographic variables such as gender and age cannot be added to the path analysis in this study. While individual characteristics are not the focus of this study, they also play a noticeable role in shaping individual subjective well-being. Perhaps we can view academic achievement as an individual’s ability, but future research should consider the influence of more individual factors on adolescents’ subjective well-being, especially those factors related to academic pressure. Third, limited by the research theme, this study did not explore whether cultural differences would affect adolescents’ subjective well-being. As the results of this study indicate that under the background of China’s college-oriented culture, the effect of family capital on adolescents’ subjective well-being through academic achievement is negative, which is different from some previous studies. In light of this, further research should be carried out in the future to consider the cultural factors to compare whether there are differences in academic pressure in different countries and whether this will lead to differences in adolescents’ subjective well-being. In addition, future research needs to consider more about the impact of individual characteristics related to academic achievement, such as academic pressure, achievement motivation and other factors on adolescents’ subjective well-being.

## Data availability statement

The original contributions presented in the study are included in the article/supplementary material, further inquiries can be directed to the corresponding author.

## Author contributions

All authors contributed to the study conception and design. XW, ZL, and YL: performed material preparation, data collection, and analysis. XW wrote the first draft of the manuscript. All authors contributed to the article and approved the submitted version.

## Conflict of interest

ZL was employed by the company Guangdong Zhongda Management Consulting Group Co., Ltd.

The remaining authors declare that the research was conducted in the absence of any commercial or financial relationships that could be construed as a potential conflict of interest.

## Publisher’s note

All claims expressed in this article are solely those of the authors and do not necessarily represent those of their affiliated organizations, or those of the publisher, the editors and the reviewers. Any product that may be evaluated in this article, or claim that may be made by its manufacturer, is not guaranteed or endorsed by the publisher.

## References

[ref1] An W. H. Western B. (2019). Social capital in the creation of cultural capital: family structure, neighborhood cohesion, and extracurricular participation. Soc. Sci. Res. 81, 192–208. doi: 10.1016/j.ssresearch.2019.03.015, PMID: 31130196

[ref2] Anglim J. Horwood S. Wood J. K. (2020). Predicting psychological and subjective well-being From personality: A meta-analysis. Psychol. Bull. 146, 279–323. doi: 10.1037/bul0000226, PMID: 31944795

[ref3] Asadullah M. N. Xiao S. Z. Yeoh E. (2018). Subjective well-being in China, 2005-2010: The role of relative income, gender, and location. China Econ. Rev. 48, 83–101. doi: 10.1016/j.chieco.2015.12.010

[ref4] Bear G. G. Yang C. Y. Huang X. S. (2018). Differences in school climate and student engagement in China and the United States. Sch. Psychol. Q. 33, 323–335. doi: 10.1037/spq0000247, PMID: 29878823

[ref5] Bourdieu P. (1973). Cultural Reproduction and Social Reproduction. London: Tavistock, 178.

[ref6] Bourdieu P. (1984). A social Critique of the Judgement of taste. Traducido del francés por R. Nice. Londres: Routledge.

[ref7] Bradley R. H. Corwyn R. F. (2002). Socioeconomic status and child development. Annu. Rev. Psychol. 53, 371–399. doi: 10.1146/annurev.psych.53.100901.135233 11752490

[ref8] Breinholt A. Jæger M. M. (2020). How does cultural capital affect educational performance: signals or skills? Br. J. Sociol. 71, 28–46. doi: 10.1111/1468-4446.12711 31903604

[ref9] Bronfenbrenner U. Morris P. A. (2007). The bioecological model of human development. *Handbook Of child*. Psychology 1, 793–828. doi: 10.1002/9780470147658.chpsy0114

[ref10] Bucker S. Nuraydin S. Luhmann M. (2018). Subjective well-being and academic achievement: a meta-analysis. J. Res. Pers. 74, 83–94. doi: 10.1016/j.jrp.2018.02.007

[ref11] Busseri M. A. (2018). Examining the structure of subjective well-being through meta-analysis of the associations among positive affect, negative affect, and life satisfaction. Personal. Individ. Differ. 122, 68–71. doi: 10.1016/j.paid.2017.10.003

[ref12] Büyükkıdık S. Bakırarar B. Bulut O. (2018). Comparing the performance of data mining methods in classifying successful students with scientific literacy in PISA 2015. In *The 6th International Congress on Measurement and Evaluation in Education and Psychology*. Prizren: Kosovo.

[ref13] Cao F. Yuan G. (2018). Influence and enlightenment of children's academic achievement, academic self-concept and happiness view on well-being: a follow-up study on students' well-being from grade 6 to grade 9. Contemp. Educ. Sci. 6, 53–63. doi: 10.3969/j.issn.1672-2221.2018.02.011

[ref14] Carolan B. V. Wasserman S. J. (2015). Does parenting style matter? Concerted cultivation, educational expectations, and the transmission of educational advantage. Sociol. Perspect. 58, 168–186. doi: 10.1177/0731121414562967

[ref15] Casas F. Gonzalez-Carrasco M. (2019). Subjective well-being decreasing with age: new research on children Over 8. Child Dev. 90, 375–394. doi: 10.1111/cdev.13133, PMID: 30106474

[ref16] Cheng S. T. Kaplowitz S. A. (2016). Family economic status, cultural capital, and academic achievement: the case of Taiwan. Int. J. Educ. Dev. 49, 271–278. doi: 10.1016/j.ijedudev.2016.04.002

[ref17] Cohen J. McCabe L. Michelli N. M. Pickeral T. (2009). School climate: research, policy, practice, and teacher education. Teach. Coll. Rec. 111, 180–213. doi: 10.1177/016146810911100108

[ref18] Coleman J. S. (1988). Social capital in the creation of human capital. Am. J. Sociol. 94, S95–S120. doi: 10.1086/228943

[ref19] Crede J. Wirthwein L. McElvany N. Steinmayr R. (2015). Adolescents’ academic achievement and life satisfaction: the role of parents’ education. Front. Psychol. 6:52. doi: 10.3389/fpsyg.2015.00052 25691877 PMC4315030

[ref20] Diener E. (1984). Subjective well-being. Psychol. Bull. 95, 542–575. doi: 10.1037/0033-2909.95.3.542 6399758

[ref21] Diener E. Oishi S. Tay L. (2018). Advances in subjective well-being research. Nat. Hum. Behav. 2, 253–260. doi: 10.1038/s41562-018-0307-6 30936533

[ref22] Du L. (2017). Adolescents' subjective well-being and its influencing factors in Beijing—based on the analysis of the two layers of linear model. Educ. Meas. Eval. 8. doi: 10.16518/j.cnki.emae.2017.07.009

[ref23] Durlak J. A. Weissberg R. P. Dymnicki A. B. Taylor R. D. Schellinger K. B. (2011). The impact of enhancing students’ social and emotional learning: A meta-analysis of school-based universal interventions. Child Dev. 82, 405–432. doi: 10.1111/j.1467-8624.2010.01564.x, PMID: 21291449

[ref24] Eichert F. Chen J. X. Torney-Purta J. (2018). Profiles of Adolescents' perceptions of democratic classroom climate and Students' influence: the effect of school and community contexts. J. Youth Adolesc. 47, 1279–1298. doi: 10.1007/s10964-018-0831-8, PMID: 29502218

[ref25] Freiberg H. J. Stein T. A. (Eds.) (1999). School Climate: Measuring, Improving and Sustaining Healthy Learning Environments. London: Falmer Press, 11–29.

[ref803] Furstenberg Jr. F. F. Hughes M. E. (1995). Social capital and successful development among at-risk youth[J]. J. Marriage Fam. 580–592.

[ref26] Gao Y. (2011). The scaling procedure in the large scale educational assessment. China Exam., 10–15. doi: 10.3969/j.issn.1005-8427.2011.11.002

[ref27] Ge M. (2015). The influence of fathers' occupation and education background on the well-being of students in the stage of primary and secondary education. Educ. Res. 20.

[ref28] Ge T. (2020). Effect of socioeconomic status on children's psychological well-being in China: the mediating role of family social capital. J. Health Psychol. 25, 1118–1127. doi: 10.1177/1359105317750462, PMID: 29278935

[ref29] Herrero I. Hughes M. (2019). When family social capital is too much of a good thing. J. Fam. Bus. Strat. 10, 100271. doi: 10.1016/j.jfbs.2019.01.001

[ref30] Hunter A. G. Chipenda-Dansokho S. Fletcher A. (2019). Social capital, parenting, and African American families. J. Child Fam. Stud. 28, 547–559. doi: 10.1007/s10826-018-1282-2

[ref31] Huston A. C. McLoyd V. C. Coll C. G. (1994). Children and poverty: issues in contemporary research. Child Dev. 65, 275–282. doi: 10.1111/j.1467-8624.1994.tb00750.x, PMID: 7516847

[ref32] Kalil A. (2015). “Inequality Begins at home: The role of Parenting in the Diverging Destinies of rich and poor children,” in Families in An Era of Increasing Inequality. eds. P. R. Amato, A. Booth, S. M. McHale, and J. Van Hook (Cham: Springer), 63–82.

[ref33] Karpudewan M. (2019). The relationships between values, belief, personal norms, and climate conserving behaviors of Malaysian primary school students. J. Clean. Prod. 237, 117748. doi: 10.1016/j.jclepro.2019.117748

[ref34] Karvonen S. Tokola K. Rimpelä A. (2018). Well-being and academic achievement: differences between schools from 2002 to 2010 in the Helsinki metropolitan area. J. Sch. Health 88, 821–829. doi: 10.1111/josh.12691, PMID: 30300928 PMC6220850

[ref35] Koch A. B. (2018). Children's perspectives on happiness and subjective well-being in preschool. Child. Soc. 32, 73–83. doi: 10.1111/chso.12225

[ref36] Konold T. Cornell D. Malone M. (2018). School climate, student engagement, and academic achievement: A latent variable. Multilevel Multi-Informant Examination. Aera Open 4:4. doi: 10.1177/2332858418815661

[ref37] La Salle T. George H. P. Evanovich L. L. (2018). An examination of school climate, victimization, and mental health problems among middle school students self-identifying With emotional and behavioral disorders. Behav. Disord. 43, 383–392. doi: 10.1177/0198742918768045

[ref38] Lampropoulou A. (2018). Personality, school, and family: what is their role in adolescents' subjective well-being. J. Adolesc. 67, 12–21. doi: 10.1016/j.adolescence.2018.05.013, PMID: 29870860

[ref39] Lee B. J. Yoo M. S. (2015). Family, school, and community correlates of children’s subjective well-being: an international comparative study. Child Indic. Res. 8, 151–175. doi: 10.1007/s12187-014-9285-z

[ref40] Letourneau N. L. Duffett-Leger L. Levac L. Watson B. Young-Morris C. (2013). Socioeconomic status and child development: a meta-analysis. J. Emot. Behav. Disord. 21, 211–224. doi: 10.1177/1063426611421007

[ref41] Li C. K. Liang Z. R. Zhang Q. (2018). Family social capital mediates the effect of poverty on children's anxiety and depression. J. Community Psychol. 46, 983–995. doi: 10.1002/jcop.22086, PMID: 30311971

[ref43] Lombardi E. Traficante D. Bettoni R. Offredi I. Giorgetti M. Vernice M. (2019). The impact of school climate on well-being experience and school engagement: A study With high-school students. Front. Psychol. 10, 2482. doi: 10.3389/fpsyg.2019.02482, PMID: 31749747 PMC6848455

[ref44] Ma X. Wilkins J. L. (2002). The development of science achievement in middle and high School: individual differences and school effects. Eval. Rev. 26, 395–417. doi: 10.1177/0193841X02026004003, PMID: 12174538

[ref45] Magnuson K. (2007). Maternal education and children's academic achievement during middle childhood. Dev. Psychol. 43, 1497–1512. doi: 10.1037/0012-1649.43.6.1497, PMID: 18020827

[ref46] Newland L. A. Giger J. T. Lawler M. J. Roh S. Brockevelt B. L. Schweinle A. (2019). Multilevel analysis of child and adolescent subjective well-being across 14 countries: child-and country-level predictors. Child Dev. 90, 395–413. doi: 10.1111/cdev.13134, PMID: 30171770

[ref801] OECD (2017). PISA 2015 Technical Report (Paris: OECD Publishing), 127–186.

[ref802] OECD (2019). PISA 2018 Results (Volume III): What School Life Means for Students’ Lives. Organisation for Economic Co-operation and Development OECD.

[ref47] Parcel T. L. Dufur M. J. Cornell Zito R. (2010). Capital at home and at school: a review and synthesis. J. Marriage Fam. 72, 828–846. doi: 10.1111/j.1741-3737.2010.00733.x

[ref48] Parcel T. L. Hendrix J. A. (2014). Family Transmission of Social and Cultural Capital. Wiley Blackwell Companion to the Sociology of Families. New York, NY: Wiley, 361–381.

[ref49] Paredes M. R. Apaolaza V. Yanez-Martinez D. (2021). The impact of the COVID-19 pandemic on subjective mental well-being: The interplay of perceived threat, future anxiety and resilience. Personal. Individ. Differ. 170:110455. doi: 10.1016/j.paid.2020.110455, PMID: 33071413 PMC7552984

[ref805] PISA (2018). PISA 2018 Database. Available at: https://www.oecd.org/pisa/data/2018database/

[ref50] Proctor C. (2014). “Subjective well-being (SWB)” in Encyclopedia of Quality of Life and Well-Being Research (Dordrecht: Springer), 6437–6441.

[ref51] Putnam R. D. (2016). Our Kids: The American Dream in Crisis. New York, NY: Simon and Schuster.

[ref52] Putwain D. W. Loderer K. Gallard D. Beaumont J. (2019). School-related subjective well-being promotes subsequent adaptability, achievement, and positive behavioural conduct. Br. J. Educ. Psychol. 90, 92–108. doi: 10.1111/bjep.12266, PMID: 30657589

[ref53] Rand K. L. Shanahan M. L. Fortney S. K. (2020). Hope and optimism as predictors of academic performance and subjective well-being in college students. Learn. Individ. Differ. 81:101906. doi: 10.1016/j.lindif.2020.101906

[ref54] Reardon S. F. (2019). Educational opportunity in early and middle childhood: using full population administrative data to study variation by place and age. RSF J. Soc. Sci. 5, 40–68. doi: 10.7758/RSF.2019.5.2.03, PMID: 31168469 PMC6545991

[ref55] Rees G. (2018). The association of childhood factors with children’s subjective well-being and emotional and behavioural difficulties at 11 years old. Child Indic. Res. 11, 1107–1129. doi: 10.1007/s12187-017-9479-2

[ref56] Ren W. Zhu X. W. Yang J. H. (2021). The SES-based difference of adolescents' digital skills and usages: an explanation from family cultural capital. Comput. Educ. 177. doi: 10.1016/j.compedu.2021.104382 [Epub ahead of print]

[ref57] Samuel R. Bergman M. M. Hupka-Brunner S. (2013). The interplay between educational achievement, occupational success, and well-being. Soc. Indic. Res. 111, 75–96. doi: 10.1007/s11205-011-9984-5

[ref58] Stahl J. F. Schober P. S. Spiess C. K. (2018). Parental socio-economic status and childcare quality: early inequalities in educational opportunity? Early Child. Res. Q. 44, 304–317. doi: 10.1016/j.ecresq.2017.10.011

[ref59] Steel P. Taras V. Bosco F. (2018). The happy culture: A theoretical, meta-analytic, and empirical review of the relationship Between culture and wealth and subjective well-being. Personal. Soc. Psychol. Rev. 22, 128–169. doi: 10.1177/1088868317721372, PMID: 28770649 PMC5892848

[ref60] Steinmayr R. Crede J. McElvany N. Wirthwein L. (2016). Subjective well-being, test anxiety, academic achievement: testing for reciprocal effects. Front. Psychol. 6:1994. doi: 10.3389/fpsyg.2015.01994 26779096 PMC4705295

[ref61] Steinmayr R. Heyder A. Wirthwein L. (2018). School-related and individual predictors of subjective well-being and academic achievement. Front. Psychol. 9, 1–13. doi: 10.3389/fpsyg.2018.02631, PMID: 30622497 PMC6308923

[ref62] Suzuki Y. Maeda N. Urabe Y. (2019). Physical activity changes and its risk factors among community-dwelling Japanese older adults during the COVID-19 epidemic: associations with subjective well-being and health-related quality of life. Rev. Gen. Psychol. 17, 458–474. doi: 10.3390/ijerph17186591 PMC7557874 32927829

[ref63] Suzuki Y. Urabe Y. (2020). Physical activity changes and its risk factors among community-dwelling Japanese older adults during the COVID-19 epidemic: associations with subjective well-being and health-related quality of life. Int. J. Environ. Res. Public Health 17. doi: 10.3390/ijerph17186591, PMID: 32927829 PMC7557874

[ref64] Thapa A. Cohen J. Guffey S. Higgins-D’Alessandro A. (2013). A review of school climate research. Rev. Educ. Res. 83, 357–385. doi: 10.3102/0034654313483907

[ref65] Villani D. Sorgente A. Antonietti A. (2019). The role of spirituality and religiosity in subjective well-being of individuals With different religious status. Front. Psychol. 10. doi: 10.3389/fpsyg.2019.01525, PMID: 31354566 PMC6630357

[ref66] Waddling J. Bertilsson E. Palme M. (2019). Struggling with capital: a Bourdieusian analysis of educational strategies among internationally mobile middle class families in Sweden. Discourse Stud. Cult. Politics Educ. 40, 697–716. doi: 10.1080/01596306.2019.1598610

[ref67] Wang M. T. Degol J. L. (2016). School climate: a review of the construct, measurement, and impact on student outcomes. Educ. Psychol. Rev. 28, 315–352. doi: 10.1007/s10648-015-9319-1

[ref68] Weeldenburg G. Borghouts L. B. Vos S. (2020). Similar but different: profiling secondary school students based on their perceived motivational climate and psychological need-based experiences in physical education. PLoS One 15, e0228859. doi: 10.1371/journal.pone.0228859, PMID: 32040543 PMC7010287

[ref69] Yang C. Y. Sharkey J. D. Dowdy E. (2018). Bullying victimization and student engagement in elementary, middle, and high schools: moderating role of school climate. Sch. Psychol. Q. 33, 54–64. doi: 10.1037/spq0000250, PMID: 29629789

[ref70] Ye Z. Wen M. Lin D. H. (2020). Subjective family socio-economic status, school social capital, and positive youth development among young adolescents in China: a multiple mediation model. Int. J. Psychol. 55, 173–181. doi: 10.1002/ijop.12583, PMID: 31066032

[ref71] Zullig K. J. Huebner E. S. Patton J. M. (2011). Relationships among school climate domains and school satisfaction. Psychol. Sch. 48, 133–145. doi: 10.1002/pits.20532

